# Cumulative live birth rates with autologous oocytes plateau with fewer number of cycles for each year of age > 42

**DOI:** 10.1186/s12958-023-01144-z

**Published:** 2023-10-23

**Authors:** David B. Seifer, Sarah F. Wang, David Frankfurter

**Affiliations:** Division of Reproductive Endocrinology and Infertility, Department of Obstetrics, Gynecology and Reproductive Sciences, Yale University School of Medicine, Yale Fertility Center 200 West Campus Drive Orange, Orange, CT USA

**Keywords:** Autologous oocytes, Cumulative live birth rates, Age > 42, Advanced reproductive age women, SART CORS

## Abstract

**Objective:**

To disaggregate the Society for Assisted Reproductive Technology Clinic Outcome Reporting System (SART CORS) age category of “ > 42” and compare age-stratified cumulative live birth rates (CLBR) > 42 years old.

**Design:**

Retrospective cohort study of autologous linked ART cycles.

**Setting:**

United States (US) National ART Database.

**Patient(s):**

Women > 42 years old without a history of prior ART cycles who underwent ART between 2014–2020 as reported to the SART CORS database.

**Intervention(s):**

Disaggregate the SART CORS age category of “ > 42” into age-stratified cumulative live birth rates (CLBR).

**Main Outcome Measure(s):**

Age-stratified cumulative live birth rates (CLBR) for women ≥ 43 years old.

**Results:**

Between 2014–2020, 24,650 women > 42 years old without history of prior ART underwent 58,132 cycles, resulting in 1,982 live births. Women ages 43, 44, 45, 46, 47, 48, 49, ≥ 50 achieved maximal CLBR of 9.7%, 8.6%, 5.0%, 3.6%, 2.5%, 1.5%, 2.7%, 1.3%, respectively. CLBR for women between 43–45 were significantly higher compared to those 46 and older (*p* < 0.05). Among women 46 and older, CLBR were not significantly different. Women ages 43 and 44 did not exhibit a significant increase in CLBR beyond the 5th cycle. Age 45 and 46 reached CLBR plateau by the 3rd cycle. Age ≥ 47 CLBR plateaued after the first cycle. After adjusting for age, race/ethnicity, BMI, nulliparity, etiology of infertility, number of oocytes retrieved, embryos transferred, blastocyst transfer, use of ICSI, PGT, and ART treatment cycle number, there was no association between markers of ovarian reserve (day 3 FSH and random AMH levels) and live birth for women > 42.

**Conclusions:**

While CLBR of autologous cycles from women 42 or younger generally plateau by cycle number 5, age-stratified cycles from women > 42 plateau after fewer cycles to maximize CLBR. Patient and physician expectations for maximum CLBR beyond 42 may be practically based on fewer planned cycles before reaching an age-specific CLBR plateau than may have been previously expected.

## Introduction

The United States lacks contemporary, publicly available, autologous oocyte assisted reproductive technology (ART) age-stratified outcome data for patients > 42 years old. After 2015, the CDC aggregated outcome data beyond age 42 or 43, depending on the year [1, 2]. Discrete, non-aggregated, age-specific outcome information is required to appropriately counsel women > 42 considering autologous ART. In 2018, 11,725 retrieval cycles, 8.7% of all U.S. retrieval cycles, were performed for patients > 42 years old [1].

While donor egg IVF, may be encouraged, many do not accept this option [2]. Attitudes regarding egg donation vary greatly based on ethnicity and religious affiliation [3]. Often, couples may feel compelled to achieve “peace of mind” by trying everything they can to conceive a biological child [2]. The drive to use one’s own eggs is further incentivized when insurance benefits cover autologous IVF and exclude donor egg IVF [4, 5]. Poor prognosis patients commonly undertake multiple attempts at IVF before giving up or transitioning to donor egg IVF [6].

There are a few studies analyzing live birth rates (LBR) in women over the age of 42 [7–10] and even fewer assessing cumulative live birth rates (CLBR) in older women. The largest of these studies examined fewer than 1300 cycles [11, 12]. In 2010, Stern et al. analyzed the Society for Assisted Reproductive Technology Clinical Outcomes Reporting System (SART CORS) database to describe the cumulative live birth rates for age groups (< 35; 35–37; 38–40; 41–42; and ≥ 43) as provided by the Centers for Disease Control (CDC) under the Fertility Clinic Success Rate and Certification Act (FCSRCA) of 1992. The CDC’s ART National Summary Reports prior to 2016 revealed an appreciable decline in pregnancy likelihood beyond age 42 which ultimately approached zero [1]. It is essential to define the point beyond which the pursuit of autologous IVF becomes futile. Unfortunately, the literature remains deficient in discrete age-specific CLBR data for women > 42 years of age. We disaggregated the SART CORS > 42-year-old designation, and compared autologous egg IVF CLBR for each year of age > 42 years to attempt to determine the point at which cycle CLBR plateaued. Plateauing was defined as the point beyond which additional ART cycles did not statistically increase CLBR.

## Material and methods

### Study design

This was a retrospective cohort study of initial (no prior history of ART) autologous linked ART cycles of women > 42 between 2014–2020 derived from the SART CORS database. Beginning in 2014 SART CORS, linked all transfers regardless of fresh or frozen to their specific index retrieval. This study was deemed exempt from review by the Yale Institutional Review Board because it relied on anonymous and de-identified data. SART CORS data were collected through voluntary submission, verified by SART, and reported to the Centers for Disease Control and Prevention (CDC) in compliance with the FCSRCA of 1992 (Public Law 102–493). In 2004, SART gained access to the SART CORS data system for the purposes of conducting research. By 2019, 81% of US IVF clinics were SART members reporting 90% of all IVF cycles in the US [13].

The data in the SART CORS are validated annually with some clinics receiving on-site visits for chart review based on an algorithm for clinic selection. During each visit, data reported by the clinic are compared with information recorded in patients’ charts. In 2021, records for 1,945 cycles at 33 clinics were randomly selected for full validation and 262 fertility preservation cycles were selected for partial validation. Nine out of ten data fields selected for validation were found to have discrepancy rates of ≤ 5% [13]. The exception was the diagnosis field, which, depending on the diagnosis, had a discrepancy rate between 0.7% and 9.1%.

### Statistical analysis

Subjects included were women > 42 years old who had no prior history of assisted reproductive technology (ART) and underwent autologous ART treatment between 2014 and 2020, as reported in the SART CORS database. A treatment cycle was defined as any intended retrieval cycle, whether cancelled or not, and all embryo transfers derived from that retrieval. Each treatment cycle for included subjects was extracted from the database. Each subject’s treatment cycle was assigned a sequential number to designate consecutive cycles. CLBR was calculated for each sequential cycle. CLBR was defined as the number of live births up to, and including, a specific cycle divided by the total number of patients undergoing the initial cycle start. This aimed to illustrate the likelihood of achieving a live birth from a specific ART treatment during the study period.

Patients were categorized into distinct age groups based on their age at the initiation of their first treatment cycle. Each age group was defined by one-year intervals up to 50 years old. Due to the limited number of subjects older than 50, those aged 50 years and above were grouped together. The sequential CLBR was examined across different age groups and within each age group. The log-rank test was employed to compare the overall CLBR patterns across all cycles between the various age groups, and chi square tests were used to compare specific CLBR between different groups. Within each age group, the 95% confidence interval difference of the mean was utilized to identify any significant differences in outcomes between consecutive treatment cycles. By determining the cycle number at which there were no significant increases in CLBR, we identified a suggested maximum number of ART attempts before considering abandoning autologous ART.

The baseline patient demographics and clinical characteristics, including markers of ovarian reserve (random day AMH, day 3 FSH) and infertility diagnoses, were examined and compared among different age groups using ANOVA and chi-square tests. Infertility diagnoses were categorized into seven distinct groups: diminished ovarian reserve (DOR), tubal factor, uterine factor, ovulatory/polycystic ovarian syndrome, endometriosis, unexplained, and male factor. These were not mutually exclusive categories and patients could have more than one of these diagnoses.

Cycle specific clinical information including number of oocytes retrieved, embryos formed, embryos transferred, percentage of cycles with blastocyst transfer, percentage of cycles using ICSI, and percentage of cycles with preimplantation genetic testing (PGT) were compared across each cycle and between age groups using ANOVA tests. Additionally, multivariable logistic regression was performed to identify significant predictors of live birth for ART treatment in women of advanced reproductive age.

## Results

Between 2014–2020, 24,650 women > 42 years old without history of prior ART underwent 54,132 treatment cycles, resulting in 1,982 live births. Overall, these 54,132 cycles represented 6.6% of the total number of ART cycles of all ages in women without history of prior ART performed during this same time period. Between 2014 and 2020 the number of women ≥ 43 years old who underwent ART increased from 3065 to 3755 (*p* < 0.05) but represented a decline from 4.8% to 4.3% (*p* < 0.01) of the total percent of women who underwent ART at any age.

**Fig. 1:**
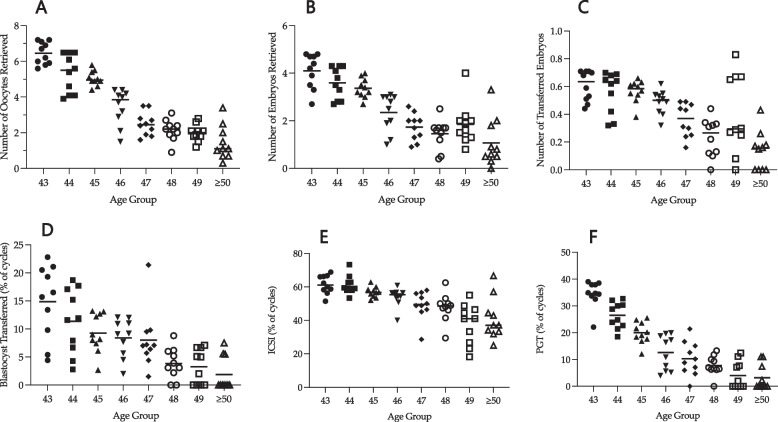
Cycle-specific clinical characteristics for all cycles according to age group. Each point denotes CLBR from each specific cycle (1 through 10). Panel **A** shows number of oocytes retrieved from each ART treatment cycle. Panel **B** shows number of embryos formed from each ART cycle. Panel **C** shows total number of embryos transferred from each ART cycle. Panel **D** shows the percentage of cycles in which a blastocyst was transferred. Panel **E** shows the percentage of cycles that used ICSI. Panel **F** shows the percentage of cycles that used PGT

Table 1 summarizes the demographic and clinical profile of the sample population for their initial cycle. The diagnosis of diminished ovarian reserve (DOR) was present in 69% of the total cycles with 93% having day 3 FSH > 10 IU/L and 71% having AMH < 1. Within each advancing age group, there was an age-specific increase in the percentage of patients with DOR, day 3 FSH > 10, and AMH < 1 (*p* < 0.001, *p* < 0.01, *p* < 0.001, respectively). Figure 1 illustrates the cycle-specific characteristics for every treatment cycle stratified by age group. There were notable differences in the number of oocytes retrieved and embryos formed across age groups for every ART treatment cycle. Specifically, for each treatment cycle, subjects aged 43 had higher numbers of oocytes retrieved and embryos formed compared to those who were 50 years or older (*p* < 0.05). Within age groups 43–47, there was a significant decrease in number of oocytes retrieved and embryos formed with each subsequent ART cycle (*p* < 0.05). For the population studied, subjects who were younger had significantly higher numbers of transferred embryos, percent blastocyst transfers, and were more likely to use ICSI and PGT (*p* < 0.001). Subjects in age groups 43 and 44 had fewer numbers of transferred embryos and percent of blastocysts transferred with each subsequent treatment cycle (*p* < 0.01). Subjects that were 45 years old also had a lower percent of blastocyst transfers with each subsequent treatment cycle (*p* < 0.05). The other age groups did not demonstrate significant differences in number of transferred embryos, or percentage of blastocyst transfers over each sequential treatment cycle.

**Table 1:**
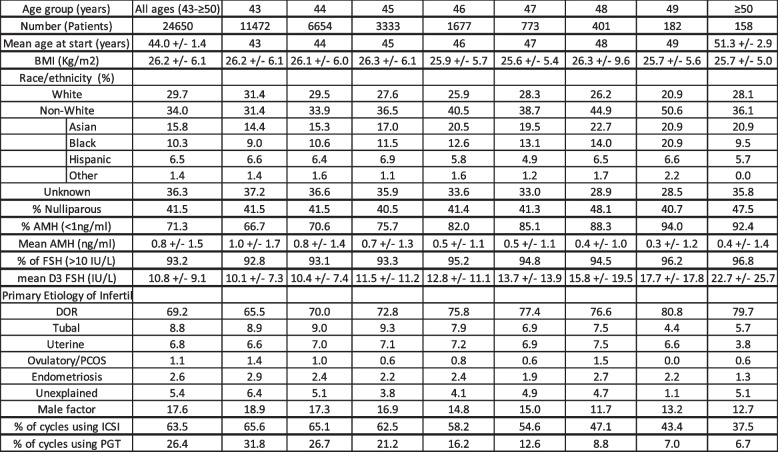
Population demographics and clinical profile for 2014-2020 cycles for initial cycle start. ** +/- **values are means ± SD. PCOS denotes polycystic ovarian syndrome. Multiple diagnoses of infertility were possible; therefore, the totals are greater than 100%

**Fig. 2:**
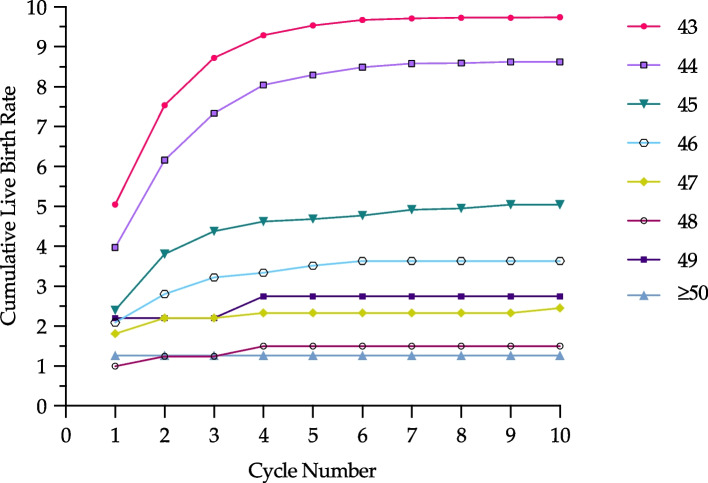
Age-specific CLBR between ages 43 and ≥ 50 years old. This shows cumulative live-birth rates over the course of sequential ART treatment cycles, stratified by age at time of first ART cycle

Figure 2 illustrates CLBR for each age > 42. CLBR significantly different between ages 43 and ≥ 50 (*p* < 0.05). Maximal CLBR was highest for 43-year-old subjects (~ 9.7%) and lowest for those ≥ 50 (1.3%); *p* < 0.001. Subjects aged 44, 45, 46, 47, 48, and 49 achieved maximal CLBR of 8.6%, 5.0%, 3.6%, 2.5%, 1.5%, and 2.7%, respectively. CLBR for subjects between 43–45 were significantly higher compared to those 46 and older (*p* < 0.05). Among subjects 46 and older, CLBR were not significantly different (*p* > 0.05).

**Fig. 3:**
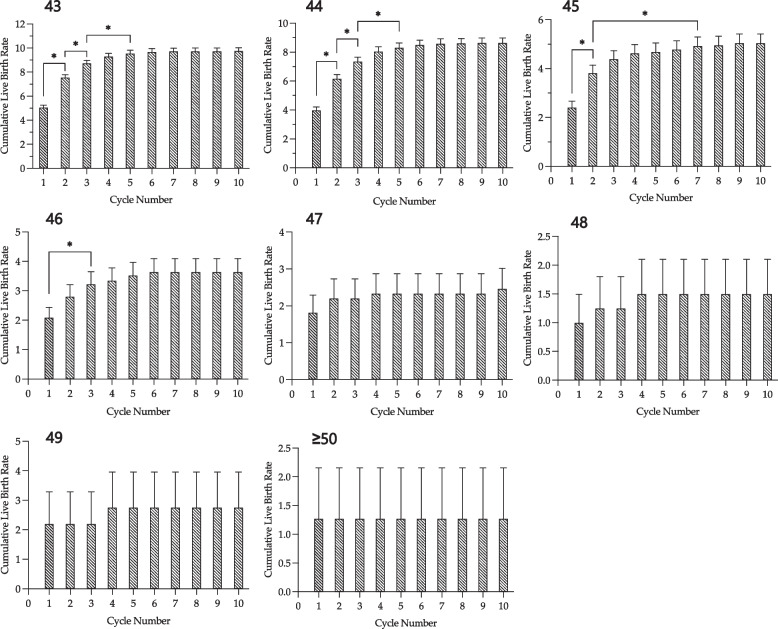
Age-specific CLBR across sequential ART treatment cycles. Each panel demonstrates age-specific CLBR according to cycle number. Asterisk (*) denotes significance with *p* < 0.05

Figure 3 demonstrates the age-specific CLBR across consecutive ART treatment cycles. For each advancing age, the CLBR reached a plateau with fewer cycles (Figs. 2 and 3, Table 2). Specifically, patients aged 43 and 44 did not exhibit a significant increase in CLBR beyond the 5th cycle. While both age groups demonstrated no significant difference in CLBR between cycles 3 and 4, there was a slight increase in CLBR between cycles 3 and 5 (0.8% and 1% respectively, *p* < 0.05). However, beyond the 5th cycle, there was minimal noticeable increase in CLBR (0.2% and 0.3% respectively, *p* > 0.05). For 45-year-old patients, the CLBR reached a plateau by the 2nd cycle, with approximately 3.8% CLBR, and the maximum CLBR of 5% was achieved by the 7th cycle. Although there was a significant increase in CLBR between cycles 2 and 7 (*p* < 0.05), the plateau was considered to be at cycle 2 since the CLBR between cycles 2–6 did not show significant differences. For patients who were 46 years old, there was no change in CLBR between cycles 1 and 2, with a CLBR of 2.1%. However, by the 3rd cycle, the CLBR increased to 3.2% (*p* < 0.05), which was higher than that observed in the first cycle. There was no significant increase in CLBR beyond cycle 3, although a maximal CLBR of ~ 3.6% was reached by the 6^th^ cycle (*p* > 0.05). For patients ≥ 47 years old, CLBR plateaued after their first cycle.

**Table 2:**
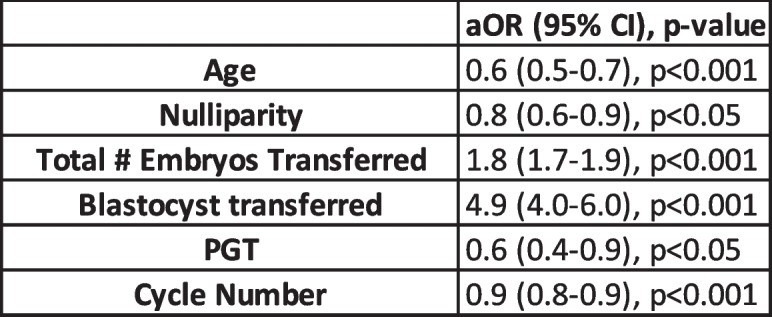
Significant Predictors of Live Birth for patients of advanced reproductive age undergoing ART. Multivariate logistic regression analysis performed adjusting for age, ethnicity, BMI, nulliparity, AMH < 1, D3FSH > 10, etiology of infertility, number of oocytes retrieved, embryos transferred, blastocyst transfer, use of ICSI, PGT, and ART treatment cycle number

Multivariable logistic regression analysis was performed to identify significant predictors of live birth for women of advanced reproductive age undergoing ART treatment cycles (Table 2). As expected, each advancing age beyond 42 and increasing number of ART treatment cycles was associated with decreased odds of live birth. Total number of embryos transferred and transfer of a blastocyst were the most significant predictors of live birth (aOR 1.8 and 4.9, respectively). Among women older than 42 years, PGT use was associated with decreased odds of live birth. After adjusting for age, race/ethnicity, BMI, nulliparity, etiology of infertility, number of oocytes retrieved, embryos transferred, blastocyst transfer, use of ICSI, PGT, and ART treatment cycle number, there was no association between markers of ovarian reserve (day 3 FSH and random AMH levels) and live birth.

## Discussion

This is the first comprehensive retrospective analysis of cumulative live birth rates stratified by age in women > 42 using the SART CORS. This study is an analysis of the first 7 years (2014–2020) of linked ART cycle data and CLBR from one of the largest standardized and validated data sets available in the United States.

It has been reported that CLBR for women 42 or younger generally plateau by the 4^th^ to 5^th^ ART cycle [14–17]. According to our analysis of the SART CORS data set, CLBRs plateau after fewer and fewer cycles for women ≥ 43. Our findings indicate that women 43 and 44 years old can be informed that CLBR is ~ 9% and 7%, respectively, however, it is unlikely that that additional live births will occur beyond three retrieval cycles. For 43 year old women, the CLBR after three retrievals was ~ 8.5% and by the fifth retrieval the CLBR reached a maximum of 9.7%. Similarly, for those 44 years old, the CLBR reached 7.5% by the third retrieval cycle with a maximum CLBR noted by cycle five at 8.6%. While a statistical difference was noted between cycles three and five for both these ages, the impact of each additional retrieval cycle was well less than 1% for each retrieval beyond the third. Therefore, the clinical utility of additional retrievals for 43 and 44 year old women is questionable.

Women 45 and 46 years old should be informed that CLBR is approximately 4% and 3%, respectively, and that each subsequent retrieval beyond the second (4% and ~ 3% respectively) affects the CLBR by < 0.5% to yield a maximum CLBR for these ages of ~ 5% and 3.6% respectively. CLBR using autologous eggs are minimal for women ≥ 47 years old, ranging from 1–2% and do not change appreciably beyond the initial cycle. The per cycle LBR is 1% or less for women over the age of 46. These observations are consistent with the fact that at age 43, blastocyst aneuploidy rates approach 85% and continue to increase with advancing age [18, 19].

Our findings reveal a clear trend of decreasing number of embryos transferred and blastocyst transfers with increasing age > 42 (Fig. 1). These factors, as indicated by our data in Table 2, emerge as the most influential predictors of live birth. Consequently, it is not surprising that the CLBR for women aged 47 or older remains relatively unchanged after the first treatment cycle. These findings are reinforced by the elevated incidence of aneuploidy observed in blastocyst biopsies from women over 44 [19]. However, it is noteworthy that preimplantation genetic testing (PGT) was associated with decreased odds of live birth in this specific population. This may be due to the observed lower utilization of PGT in older age groups and the limited availability of embryos for testing. For women 43 and older, we noted that markers of ovarian reserve did not appear to be predictive of live birth despite prior findings looking at aggregated age group data (> 42) suggesting the contrary [20]. This is likely due to the fact that AMH values are so low in these advanced age groups that it loses its predictive ability.

The current analysis of this large dataset helps frame expectations for maximum CLBR beyond age 42. Our data suggests that women > 42 years old reach a maximum CLBR after 1–5 cycle retrieval cycles, depending on their specific age. For those who do not yield transferable embryos, the value of IVF with autologous eggs could be questioned after fewer attempts. Because all transfer attempts are linked to a single retrieval, each retrieval may yield multiple transfer attempts and therefore number of “cycles” to achieve a live birth may be underrepresented in this study. Accordingly, our findings add credence to the need for early counseling regarding alternatives to autologous ART (donor egg or adoption) when it comes to the decision-making process attempts at autologous ART for women ≥ 44 years of age.

The CLBRs presented here are based on cycle starts. The relatively high cancellation rates reported in the setting of autologous eggs beyond age 40 predict lower CLBR [9]. The ASRM defines futility as ≤ 1% probability for pregnancy [21]. Considering the data presented here, autologous IVF can be considered futile beyond the first attempt at ages 46 and 47 and with essentially any attempt for women older than 47. This is consistent with the findings of Gunnala et al. who examined IVF cycles in women ≥ 45 [9]. They reported that approximately 40% of cycles could either not start due to findings at baseline or were cancelled prior to retrieval. For those going to transfer, LBR was 3.4%. Others have reported on the futility of LBRs in women 45 years and older [10, 11, 22]. Our findings are more comprehensive than, but remain consistent with a prior report using the SART CORS database [16].

Diminished ovarian reserve is an inevitable part of the human experience. While there have been anthropological rationalizations for the process, they do not console those who are faced, late in life, with the desire to conceive [23, 24]. We were left with a precipitous decline in fecundity during the fifth decade of life [25]. Contemporary population-based data for specific ages are lacking, but grouped data mirror our findings. In 2022, the National Center for Health Statistics reported that the annual provisional birth rate for women between 40-44 was 12.5 births per 1000 women compared to 1.1 births per 1000 women between ages 45-49 [26, 27]. While these data do not allow for a direct comparison to ART outcomes, they demonstrate that birth rates in the general population and the ART population become more comparable beyond age 45. Currently there remain no clear and proven therapeutic options for clock reversal, ovarian regeneration, or oocyte replenishment. What remains, is the application of a technology that has proven success for youthful individuals to a population with little demonstrable benefit [8]. We provide a representative guide, derived from a large and validated contemporary data set, to allow women in their mid- to late-forties to make informed decisions about their reproductive options. Patient centered care requires a clear understanding of the likelihood of the desired health outcome. It is incumbent on care providers to counsel, and for patients to be fully informed, when the probability of live birth is exceedingly low. Such counseling requires data and neither speculation nor a potentially biased opinion. Our data allow for the provision of such patient centered care and the discussion of realistic patient and physician expectations in the context of women age 43 and older.

Our study is limited by its retrospective nature. Prospective controlled trials assessing reproductive outcomes at the twilight of reproductive potential are rare and limited. Morgia et al. conducted an RCT to evaluate different COH regimens and noted low implantation rates for women > 40, however, women > 43 were not included [28]. Such center specific reporting is bound to be limited by small sample size and lack of power. The paucity of controlled IVF trials for extremely advanced maternal age is likely due to limited resources and the relatively small percentage of patients in these age groups. Reporting is thereby limited to large retrospective datasets. Our study is strengthened by its sample size, the largest involving these age ranges to date, and its reliance on a validated national data set of contemporary ART practices. Using the SART CORS database, we were able to discreetly examine the potential for cumulative live birth, arguably the most meaningful IVF outcome, at the terminal boundary of reproductive life.

We show that beyond age 45 live birth outcomes become quite rare. Meager odds may not dissuade some, but it is incumbent upon providers to be able to provide realistic likelihoods for desired goals. Vaguely stating low odds or providing wide ranges cannot suffice as satisfactory when stakes and costs are as high as they are for a single ART cycle. There remain circumstances when cost to the patient is not an issue, as when insurance coverage is guaranteed no matter the prognosis. In such situations, when treatment becomes futile, there is a clear need for objective data as presented here. As noted by ASRM, not offering treatment in the context of futility is an appropriate expression of professional integrity [21]. Stopping treatment may run counter to the patient’s desire. However, cessation of treatment may remain justified because continuing can prevent patients from clearly addressing other options. This is an area that needs to be consistent across an IVF center and not appear arbitrary [21].

## Data Availability

Data is available upon request to authors.
